# Genome sequence of a dissimilatory Fe(III)-reducing bacterium *Geobacter soli* type strain GSS01^T^

**DOI:** 10.1186/s40793-015-0117-7

**Published:** 2015-12-02

**Authors:** Guiqin Yang, Shanshan Chen, Shungui Zhou, Yongfeng Liu

**Affiliations:** Guangzhou Institute of Geochemistry, Chinese Academy of Sciences, Guangzhou, 510640 China; Guangdong Key Laboratory of Agricultural Environment Pollution Integrated Control, Guangdong Institute of Eco-Environmental and Soil Sciences, Guangzhou, 510650 China; University of Chinese Academy of Sciences, Beijing, 100049 China; BGI-Shenzhen, Main Building 11/F, Beishan Industrial Zone, Yantian District, Shenzhen, 518083 China

**Keywords:** *Geobacter soli*, Extracellular electron transfer, Insoluble Fe(III) oxides reduction, Cytochrome, Pilin protein

## Abstract

Strain GSS01^T^ (=KCTC 4545=MCCC 1 K00269) is the type strain of the species *Geobacter soli. G. soli* strain GSS01^T^ is of interest due to its ability to reduce insoluble Fe(III) oxides with a wide range of electron donors. Here we describe some key features of this strain, together with the whole genome sequence and annotation. The genome of size 3,657,100 bp contains 3229 protein-coding and 54 RNA genes, including 2 16S rRNA genes. The genome of strain GSS01^T^contains 76 predicted cytochrome genes, 24 pilus assembly protein genes and several other genes, which were proposed to be related to the reduction of insoluble Fe(III) oxides. The genes associated with the electron donors and acceptors of strain GSS01^T^ were predicted in the genome. Information gained from its sequence will be relevant to the future elucidation of extracellular electron transfer mechanism during the reduction of Fe(III) oxides.

**Electronic supplementary material:**

The online version of this article (doi:10.1186/s40793-015-0117-7) contains supplementary material, which is available to authorized users.

## Introduction

*Geobacter* is the type genus of the family *Geobacteraceae* in the order *Desulfuromonadales* within the class *Deltaproteobacteria* [1]. It currently contains 19 validly named species and 2 subspecies isolated from various environments, mostly subsurface anoxic environments. Members of the genus *Geobacter* are anaerobic, Gram-negative and rod-shaped bacteria. The *Geobacter* species have the ability to effectively transfer electrons directly onto insoluble extracellular metal (iron) oxides, and thus commonly, are the most abundant microorganisms in anaerobic soils and sediments where metal reduction is an important process [2].

*Geobacter soli* strain GSS01^T^ (=KCTC 4545=MCCC 1 K00269), is the type strain of the species *Geobacter soli* [3]. It was originally isolated from soil of an underground ancient forest in Longfu Town, Sihui City, Guangdong Province, China (23^o^ 22′ N 112^o^ 42′ E). Within the genus *Geobacter*, *G. soli* has been proposed to form a subclade together with *G. sulfurreducens* PCA, demonstrating 98.3 % similarity between the 16S rRNA gene sequences [3]. Here, we summarize the physiological features together with the whole genome sequence, annotation and data analysis of *G. soli* strain GSS01^T^.

## Organism information

### Classification and features

**Fig. 1:**
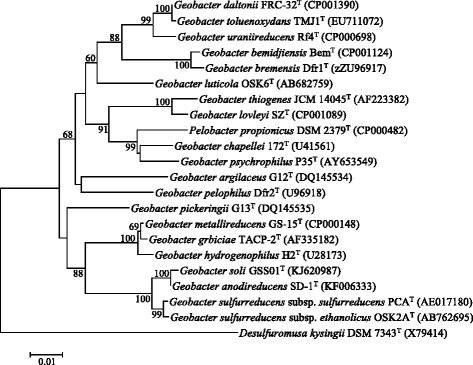
Phylogenetic tree based on 16S rRNA gene sequences showing the position of *G. soli* GSS01^T^ relative to the type strains of other species within the genus *Geobacter*. The strains and their corresponding GenBank accession numbers of 16S rRNA genes were indicated in parentheses. The sequences were aligned using Clustal W and theneighbor-joining tree was constructed based on kimura 2-paramenter distance model by using MEGA 6 [35]. Bootstrap values above 60 % were shown obtained from 1000 bootstrapreplications. Bar, 0.01 substitutions per nucleotide position. *Desulfuromusa kysingii* DSM7343 (X79414) was used as an outgroup

Based on the 16S rRNA gene phylogeny and phenotypic characteristics, strain GSS01^T^ was classified as a member of the genus *Geobacter*, showing the highest similarity to *G. anodireducens* SD-1^T^ (99.8 %) among all the type strains of the genus *Geobacter*. *G. anodireducens* was a new species which was established at almost the same time with *G. soli* [4], and therefore, no comparison was made between strains GSS01^T^ and SD-1^T^ under the same conditions*.* The high 16S rRNA gene sequences similarity of 99.8 % between these two species indicates a possibility that *G. soli* is a heterotypic synonym of *G. anodireducens*. A 16S rRNA gene-based phylogenetic tree reconstructed using the neighbor-joining method (Fig. 1) shows the phylogenetic neighborhood of *G. soli*.Within the genus *Geobacter*, *G. soli* forms a distinct subclade together with *G. anodireducens*, *G. sulfurreducens* subsp. *sulfurreducens* and *G. sulfurreducens* subsp. *ethanolicus*.

**Fig. 2:**
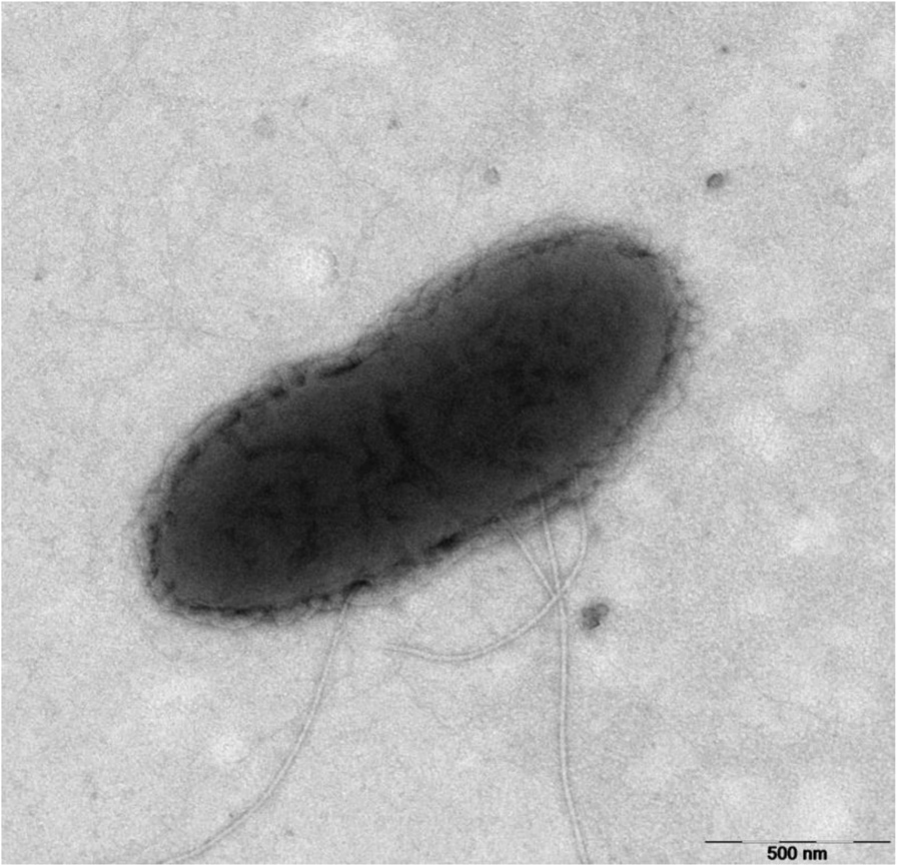
Transmission electron microscopy of strain GSS01^T^. Scale bar corresponds to 500 nm

*Geobacter soli* GSS01^T^ is anaerobic, Gram-stain-negative, motile, rod-shaped (1.0–1.7 μm in length and 0.5 μm in width) and produces monolateral flagella when grown with acetate and Fe(III) citrate (Fig. 2). Growth occurs at 16–40 °C with optimal growth at 30 °C (Table 1). With acetate as the electron donor, ferrihydrite, Fe(III) citrate, Mn (IV), sulfur and 2, 6-anthraquinone-disulphonate can be utilized as electron acceptors. With ferrihydrite as the electron acceptor, acetate, ethanol, glucose, butyrate, pyruvate, benzoate, benzaldehyde, *m*-cresol and phenol can be utilized as electron donors.

**Table 1:** Classification and general features of *G. soli* GSS01^T^ according to the MIGS recommendations [36]

MIGS Id	Property	Term	Evidence code^a^
	Classification	Domain Bacteria	TAS [37]
		Phylum *Proteobacteria*	TAS [38]
		Class *Deltaproteobacteria*	TAS [39, 40]
		Order *Desulfuromonadales*	TAS [39, 41]
		Family *Geobacteraceae*	TAS [39, 42, 43]
		Genus *Geobacter*	TAS [1, 44]
		Species *Geobacter soli*	TAS [3]
		Type strain GSS01=KCTC 4545=MCCC 1 K00269	TAS [3]
	Gram stain	Negative	TAS [3]
	Cell shape	Rod	TAS [3]
	Motility	Motile	TAS [3]
	Sporulation	Nonsporulating	TAS [3]
	Temperature range	16–40 °C	TAS [3]
	Optimum temperature	30 °C	TAS [3]
	pH range; Optimum	6–8.5; 7.0	NAS
	Carbon source	Acetate, ethanol, glucose, lactate, butyrate, pyruvate, benzoate, benzaldehyde, *m*-cresol and phenol	TAS [3]
	Terminal electron acceptor	Ferrihydrite, Fe(III) citrate, Mn(IV), sulfur, and AQDS	TAS [3]
MIGS-6	Habitat	Forest soil	TAS [3]
MIGS-6.3	Salinity	0–1.5 % NaCl (*w/v*)	NAS
MIGS-22	Oxygen requirement	Obligately anaerobic	TAS [3]
MIGS-15	Biotic relationship	Free living	NAS
MIGS-14	Pathogenicity	None known	
MIGS-4	Geographic location	Longfu Town, Sihui City, Guangdong Province, China	TAS [3]
MIGS-5	Sample collection	Mar 14, 2013	NAS
MIGS-4.1	Latitude	23.37^o^ N	TAS [3]
MIGS-4.2	Longitude	112.70° E	TAS [3]
MIGS-4.4	Altitude	11 m	NAS

^a^ *Evidence code-IDA* Inferred from direct assay, *TAS* Traceable author statement (i.e., a direct report exists in the literature), *NAS* Non-traceable author statement (i.e., not directly observed for the living, isolated sample, but based on a generally accepted property for the species, or anecdotal evidence). These evidence codes are from the Gene Ontology Project [45]

## Genome sequencing information

### Genome project history

*Geobacter soli* GSS01^T^ was selected for genome sequencing based on its phylogenetic position and its ability to reduce insoluble Fe(III) oxides with a wide range of electron donors. The genome sequence was deposited at DDBJ/EMBL/GenBank under the accession JXBL00000000. The version described in this paper is version JXBL01000000. A summary of the project and the Minimum Information about a Genome Sequence were shown in Table 2 and Additional file 1: Table S1.

**Table 2:** Project information

MIGS Id	Property	Term
MIGS-31	Finishing quality	High-quality draft
MIGS-28	Libraries used	Two libraries 463 bp PCR-free library, 6712 bp index library
MIGS-29	Sequencing platforms	Illumina Hiseq 2000
MIGS-31.2	Fold coverage	165×
MIGS-30	Assemblers	SOAP*denovo* 2.04 [5]
MIGS-32	Gene calling method	Glimmer 3.02 [9]
	Locus Tag	SE37
	Genbank ID	JXBL00000000
	Genbank Date of Release	Jan 8, 2015
	GOLD ID	Gp0109882
	Bioproject	PRJNA271628
MIGS-13	Source Material Identifier	KCTC 4545
	Project relevance	Type strain, environmental, insoluble Fe(III) oxides reduction

### Growth conditions and genomic DNA preparation

*Geobacter soli* strain GSS01^T^ was anaerobically cultivated in a mineral salts medium [MSM, containing (L^−1^) 0.6 g NaH_2_PO_4_, 0.25 g NH_4_Cl, 0.1 g KCl, 2.5 g NaHCO_3_, 10.0 ml vitamin stock solution and 10.0 ml mineral stock solution [5], pH 7.2] supplemented with 50 mM Fe(III) citrate and 10 mM acetate as the electron acceptor and donor, respectively. Total genomic DNA was extracted using a DNA extraction kit (Aidlab). The quality and quantity of the genomic DNA was determined by 0.6 % agarose gel electrophoresis with λ-Hind III digest DNA marker and by a Qubit fluorometer (Invitrogen, CA, USA) with Qubit dsDNA BR Assay kit. About 50.22 μg DNA with a concentration of 91.3 ng/μl was obtained.

### Genome sequencing and assembly

The genome of strain GSS01^T^ was sequenced at the BGI in Shenzhen using the HiSeq2000 system (Illumina, USA). Two libraries with insert size 463 bp and 6712 bp were constructed and a total of 461 Mb and 232 Mb raw data were produced before filtering, respectively. After removing the adapter, duplicated reads and short inserts from the data of large library, there remained 401 Mb and 202 Mb clean data for assembling, respectively. Then these sequences were assembled into 15 contigs using the SOAP*denovo* 2.04 [6] with K setting at 83.

### Genome annotation

Whole genomic tRNA were identified using tRNAscan (version 1.23) [7] with the bacterial model, rRNAs were found by rRNAmmer (version 1.2) [8], and sRNA were predicted using Infernal software and the Rfam database (version 10.1) [9]. The genes in the assembled genome were identified using Glimmer (version 3.02) [10]. The predicted ORFs were translated and used to search KEGG (version: 59), COG (version: 20090331), SwissPort (version: 201206), NR (version: 20121005) and GO (version: 1.419) databases. These data sources were combined to assert a product description for each predicted protein. Genes with signal peptides and transmembrane helices were predicted using SignalP server v.4.1 [11] and TMHMM server v.2.0 [12], respectively.

## Genome properties

**Fig. 3:**
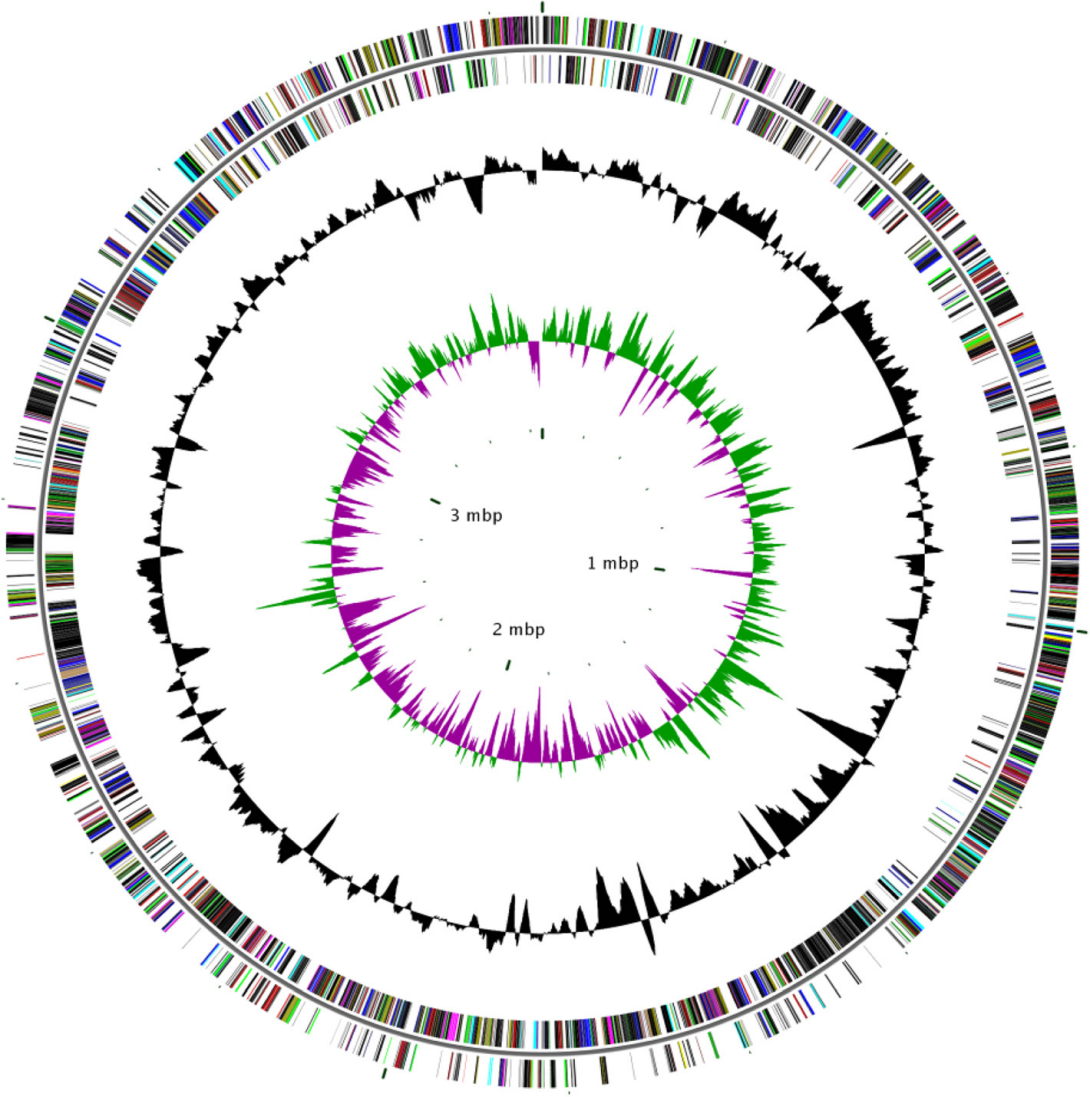
Circular map of the chromosome of *G. soli* GSS01^T^. Labeling from the outside to the inside circle: ORFs on the forward strand (colored by COG categories), ORFs on the reverse strand (colored by COG categories), RNA genes, G+C content (peaks out/inside the circle indicate values higher or lower than the average G+C content, respectively) and GC skew

The genome comprised a circular chromosome with a length of 3,657,100 bp with a GC content of 61.76 % (Fig. 3 and Table 3). It was assembled into 15 contigs. A total of 3312 genes were predicted, including 3229 protein-coding genes and 54 RNA genes (48 tRNA genes and two copies of 16S-23S-5S rRNA gene operons). Of the protein-coding genes, 1727 were assigned to COG functional categories. The detailed properties and the statistics of the genome were presented in Table 3. The distribution of genes into COG functional categories was summarized in Fig. 3 and Table 4. The nine *Geobacter* species genomes (including *G. soli*) of characterized isolates were compared in Table 5.

**Table 3:** Genome statistics of *G. soli* strain GSS01^T^

Attribute	Genome (total)	
	Value	% of total^a^
Genome size (bp)	3,657,100	100.00
DNA coding (bp)	3,293,088	90.04
DNA G + C (bp)	2,258,691	61.76
DNA scaffolds	8	
Total genes	3312	100.00
Protein coding genes	3229	97.49
RNA genes	54	1.63
Pseudo genes	28	0.84
Genes in internal clusters	79	2.39
Genes with function prediction	2626	79.29
Genes assigned to COGs	1727	52.14
Genes with Pfam domains	2693	81.31
Genes with signal peptides	257	7.76
Genes with transmembrane helices (≥3)	303	9.15
CRISPR repeats	1	

^a^The total is based on either the size of the genome in base pairs or the total number of protein coding genes in the annotated genome

**Table 4:** Number of genes associated with the 25 general COG functional categories

Code	Value	% of total^a^	Description
J	145	4.58	Translation
A	-	-	RNA processing and modification
K	73	2.31	Transcription
L	83	2.62	Replication, recombination and repair
B	1	0.03	Chromatin structure and dynamics
D	17	0.54	Cell cycle control, mitosis and meiosis
Y	-	-	Nuclear structure
V	36	1.14	Defense mechanisms
T	141	4.45	Signal transduction mechanisms
M	97	3.06	Cell wall/membrane biogenesis
N	65	2.05	Cell motility
Z	-	-	Cytoskeleton
W	-	-	Extracellular structures
U	45	1.42	Intracellular trafficking and secretion
O	80	2.53	Posttranslational modification, protein turnover, chaperones
C	153	4.83	Energy production and conversion
G	66	2.08	Carbohydrate transport and metabolism
E	162	5.12	Amino acid transport and metabolism
F	46	1.45	Nucleotide transport and metabolism
H	98	3.10	Coenzyme transport and metabolism
I	52	1.64	Lipid transport and metabolism
P	82	2.59	Inorganic ion transport and metabolism
Q	28	0.88	Secondary metabolites biosynthesis, transport and catabolism
R	157	4.96	General function prediction only
S	100	3.16	Function unknown
-	1439	45.45	Not in COGs

^a^The total is based on the total number of protein coding genes in the annotated genome

**Table 5:** Genome statistics comparison among characterized *Geobacter* species^a^

Genome name	1			2		3	4	5	6	7	8	9
	PCA	KN400	AM-1	GS-15	RCH3	Bem	SZ	Rf4	FRC-32	R1	G13	GSS01
Genome size (Mb)	3.8	3.7	4.7	4.0	3.8	4.6	3.9	5.1	4.3	4.7	3.6	3.7
G + C content (%)	60.9	61.0	60.2	59.5	59.8	60.3	54.8	54.2	53.50	60.30	62.3	61.8
Total genes	3711	3610	4171	3608	3412	4055	3605	4506	3828	4146	3298	3312
Protein-coding genes	3430	3289	4088	3520	3317	3942	3535	4417	3750	4058	3197	3229
RNA genes	240	296	57	70	51	71	53	58	57	60	61	55
Pseudogenes	41	25	26	18	44	42	18	31	21	28	40	28
Frameshifted genes	ND	16	9	12	34	35	9	21	13	19	36	21
CRISPR	ND	1	4	1	1	-	1	2	1	-	2	1

^a^the *Geobacter* species are numbered as: 1, *G. sulfurreducens*; 2, *G. metallireducens*; 3, *G. bemidjiensis*; 4, *G. lovley*; 5, *G. uraniireducens*; 6, *G. daltonii*; 7, *G. bremensis*; 8, *G. pickeringii*; 9, *G. soli*

## Insights from the genome sequence

### Genes associated with insoluble Fe(III) oxide reduction

The ability of *Geobacter* to reduce insoluble Fe(III) oxides is presumably due to the presence of a vast network of *c*-type cytochromes that transfer the electrons out of the inner membrane, through the periplasm and outer membrane to Fe(III) oxides [13, 14]. Previous reports revealed that 38 % of the *c*-type cytochrome proteins encoded in the genome of *G. sulfurreducens* were predicted to involve in the extracellular electron transfer during the reduction of insoluble Fe(III) oxides, which emphasized the importance of the *c*-type chrochromes for extracellular electron transfer [13]. Since the *c*-type cytochromes are not well conserved among *Geobacter* species [15], it is valuable to investigate the cytochrome content of different *Geobacter* species.

Proteins were considered as *c*-type cytochromes if their sequence contained at least one CXXCH motif for covalent heme binding (where X can be anything except one of [CFHPW]) [16]. The analysis of the genome of strain GSS01^T^ showed that 104 open reading frames (ORFs) in the genome contained at least one occurrence of the motif indicating that these may be cytochromes. Since this definition of cytochrome was minimal, a more stringent definition was created using sequence profiles described in the protein database InterPro as *c*-type cytochromes. Proteins were considered to be *c*-type cytochromes if their sequence contained at least one profile match in the InterPro database and at least one CXXCH motif. Results showed that genome of strain GSS01^T^ contained 76 cytochromes, and 82 % of the cytochromes contain more than one heme motif –with 7.6 hemes per cytochrome on average (Table 6). These results were in accordance with the reported data of other six *Geobacter* genomes [15].

**Table 6:** Description of the predicted cytochrome *C* proteins

Protein Id	Size/aa	Motif count	Protein Id	Size/aa	Motif count
SE37_00200	346	2	SE37_11335	488	5
SE37_00350	233	2	SE37_11345	189	6
SE37_00575	134	3	SE37_11360	467	1
SE37_00770	283	1	SE37_11370	345	2
SE37_00775	329	12	SE37_11705	353	6
SE37_00785	349	6	SE37_11710	884	27
SE37_00875	91	3	SE37_11760	2803	24
SE37_00895	330	7	SE37_11765	979	16
SE37_00905	232	3	SE37_11830	1046	22
SE37_01170	285	11	SE37_11835	279	3
SE37_01435	1206	12	SE37_11905	94	1
SE37_01700	111	1	SE37_11920	266	4
SE37_01720	231	8	SE37_11940	421	9
SE37_01740	231	8	SE37_11945	623	11
SE37_01745	747	10	SE37_11955	324	5
SE37_02090	310	11	SE37_12855	591	7
SE37_02100	155	3	SE37_12935	488	4
SE37_02785	141	1	SE37_12940	154	3
SE37_02825	430	6	SE37_13340	507	5
SE37_02835	437	6	SE37_14005	93	2
SE37_02870	426	14	SE37_14185	160	4
SE37_03120	104	1	SE37_14295	187	1
SE37_03555	482	8	SE37_15065	102	1
SE37_03965	709	27	SE37_15075	115	1
SE37_03990	456	7	SE37_15310	304	1
SE37_04285	472	5	SE37_15520	619	9
SE37_04675	343	11	SE37_16085	533	6
SE37_05760	296	3	SE37_16115	91	3
SE37_05765	363	7	SE37_16120	95	3
SE37_05900	486	3	SE37_00890	337	5
SE37_05905	90	3	SE37_01425	427	6
SE37_06935	270	4	SE37_02105	229	6
SE37_07370	419	2	SE37_02820	434	6
SE37_08180	567	1	SE37_02865	646	22
SE37_08440	478	7	SE37_09635	248	1
SE37_08660	155	1	SE37_15715	140	1
SE37_08880	337	8	SE37_16455	231	8
SE37_09795	92	3	SE37_16460	747	10

Studies with *G. sulfurreducens* have confirmed that the pili of *G. sulfurreducens* are electrically conductive and, thus, have been proposed to serve as an electronic conduit or ‘nanowire’ between the cell and the insoluble Fe(III) oxides [17]. The pili of *G. sulfurreducens* are an assembly of a pilin subunit with the conserved N-terminal sequence of bacterial type Iva pillins [17]. The genome sequence of strain GSS01^T^ contained 24 ORFs predicted to code for pilus assembly proteins (Table 7). One of them (SE37_07695) contains the conserved amino-terminal amino acid characteristic of type IVa pilins [18], and shares 85 % similarity with the pilin or PilA peptide of *G. sulfurreducens* (Gsu1496). Similar to the PilA sequence of *G. sulfurreducens* and other *Geobacter* memebers, the predicted length of SE37_07695 (75 amino acids) was considerably shorter than other bacterial pilins [17]. The N-terminal region of SE37_07695 was conserved with those of other reported PilA sequences (Additional file 2: Figure S1) and SE37_07695 contained hydrophobic amino acids (51 amino acids) predicted to form an *α*-helix using PredictProtein (https://www.predictprotein.org/). This *α*-helix has been proposed to mediate pilin-pilin interactions during assembly to form a hydrophobic filament core [19]. As observed in the pilin of *G. sulfurreducens* [17], SE37_07695 contained 6 aromatic amino acids. Five of these aromatic residues are required for pilus conductivity [20]. Besides *pilA*, almost all genes attributed to pilus biogenesis in *G. soli* have orthologs in *G. sulfurreducens**,* and the homologues of genes for the formation and assembly of pili are upstream of the *G. soli**pilA* gene, in a conserved genetic arrangement similar to that of the pili genes in *G. sulfurreducens* [17].

**Table 7:** Description of pillus assembly protein

Protein Id	Size/aa	Predicted function	Closest relatives		
			Organism	Identity	Accession no.
SE37_00040	549	Pilus assembly protein PilB	*Geobacter sulfurreducens*	95 %	WP_010941104
SE37_02260	645	Pilus assembly protein PilB	*Geobacter sulfurreducens*	97 %	WP_010943246
SE37_04495	351	Pilus assembly protein PilM	*Geobacter sulfurreducens*	97 %	WP_010942675
SE37_04505	198	Pilus assembly protein PilO	*Geobacter sulfurreducens*	95 %	WP_010942673
SE37_04510	181	Pilus assembly protein PilP	*Geobacter sulfurreducens*	84 %	WP_010942672
SE37_04515	895	Pilus assembly protein PilQ	*Geobacter sulfurreducens*	87 %	WP_010942671
SE37_05780	574	Pilus assembly protein PilB	*Geobacter sulfurreducens*	97 %	WP_010942427
SE37_05785	308	Pilus assembly protein PilM	*Geobacter sulfurreducens*	87 %	WP_010942426
SE37_05795	181	Pilus assembly protein PilO	*Geobacter sulfurreducens*	96 %	WP_010942424
SE37_07695	75	Pilus assembly protein PilA	*Pelobacter propionicus*	92 %	WP_011735547
SE37_07710	405	Pilus assembly protein PilC	*Geobacter metallireducens*	88 %	WP_004513113
SE37_08820	332	Pilus assembly protein PilZ	*Geobacter sulfurreducens*	95 %	WP_010941896
SE37_09045	123	Pilus assembly protein PilZ	*Geobacter sulfurreducens*	88 %	WP_010941849
SE37_09615	1014	Pilus assembly protein PilY	*Geobacter sulfurreducens*	93 %	WP_010941727
SE37_09620	172	Pilus assembly protein PilX	*Geobacter sulfurreducens*	98 %	WP_010941726
SE37_09625	358	Pilus assembly protein PilW	*Geobacter sulfurreducens*	94 %	WP_010941725
SE37_09630	132	Pilus assembly protein PilV	*Geobacter sulfurreducens*	86 %	WP_010941724
SE37_09690	113	Pilus assembly protein PilZ	*Geobacter sulfurreducens*	88 %	WP_010941712
SE37_10125	244	Pilus assembly protein PilZ	*Geobacter sulfurreducens*	89 %	WP_010941611
SE37_12375	267	Pilus assembly protein PilZ	*Geobacter sulfurreducens*	94 %	WP_014552065
SE37_13845	232	Pilus assembly protein PilZ	*Geobacter sulfurreducens*	91 %	WP_010940813
SE37_14140	113	Pilus assembly protein PilZ	*Geobacter sulfurreducens*	96 %	WP_010940755
SE37_15860	119	Pilus assembly protein pilZ	*Geobacter sulfurreducens*	98 %	WP_010940980
SE37_04460	196	Pilus assembly protein PilE	*Geobacter sulfurreducens*	74 %	WP_010942683

Another gene encoding putative menaquinol oxidoreductase (SE37_00765) that might be involved in the reduction of Fe(III) oxides revealed 86 % similarity to the menaquinol oxidoreducatase complex Cbc5 of *G. sulfurreducens*. The putative complex Cbc5 was an essential protein for reduction of insoluble Fe(III) oxides in both *G. sulfurreducens* and *G. uraniireducens* [13]. In addition, the existence of a number of chemotaxis proteins (count 85) and flagella proteins (count 42) in the genome of strain GSS01^T^ indicated the possibility of accessing insoluble Fe(III) oxides by chemotaxis which was only reported in *G. metallireducens* [21]. Chemotaxis, mainly depends on motility by flagella, is beneficial for Fe(III) oxide reduction [22], and deletion of the flagellin protein-encoding gene *fliC* resulted in the loss of ability to reduce insoluble Fe(III) oxides in a *G. metallireducens* strain [23].

### Reduction of other electron acceptors

*G. sulfurreducens* is capable of oxygen respiration [24] using a cytochrome *caa*_3_ oxidase complex (*coxACDB* genes) [25], which is also found in *G. soli* GSS01^T^ (SE37_15290, SE37_15295, SE37_15300, SE37_15305, SE37_15310 and SE37_15315). In addition, the *G. soli* genome contains a pair of genes encoding cytochrome *bd* quinol oxidases (SE37_06965 and SE37_06970), which is closely related to its counterparts in *G. sulfurreducens*. The presence of these proteins indicates that strain GSS01^T^ may grow with oxygen as a terminal electron acceptor. To detoxify reactive oxygen species (ROS) that produced from the oxygen respiration, *G. soli* possesses a desulfoferrodoxin (SE37_01515), a superoxide dismutase (SE37_09185), a catalase (SE37_11355), 2 peroxiredoxins (SE37_10345 and SE37_14390), 3 rubrerythrins (SE37_02245, SE37_11375 and SE37_11415), and 5 peroxidases (SE37_00200, SE37_10685, SE37_11370, SE37_13285 and SE37_16060), which were also present in *G. sulfurreducens*. Overall, the genome annotation indicates that, strain GSS01^T^ has evolved to cope with many kinds of ROS to survive oxidative stress, which can ensure cells survive in oxic environments.

Sulfate and nitrate are common electron acceptors in the anaerobic bacteria. *G. soli* possesses the essential proteins in complete pathway of assimilatory sulfate reduction, including sulfate transporter (SE37_02365, SE37_04150, SE37_08380, SE37_08385 and SE37_08390 and SE37_08640), sulfate adenylyltransferase (SE37_06140), adenylylsulfate reductase (SE37_06145) and sulfite reductase (SE37_08370, SE37_13370 and SE37_15590). Nitrate can be reduced by *G. metallireducens* but cannot be utilized by *G. sulfurreducens* [26]. Like *G. sulfurreducens*, *G. soli* contains two putative copies of periplasmic nitrite reductases: the first, NfrA (SE37_12935=GSU3154; SE37_16085 = GSU0357), is responsible for the reduction of nitrite to ammonia; the second, NrfH (SE37_12940 = GSU3155), is the small subunit whose likely role is to mediate between the quinone pool and the nitrite reductase. The nitrite reductase (NADH) small subunit, NirD (SE37_02720 = GSU2527) is also found. The presence of these ORFs and the absence of the nitrate reductase indicate the possibility that nitrite can be utilized as an electron acceptor but nitrate can be not. In addition, the putative nitric-oxide reductase NorB (SE37_00345) and NorC (SE37_00350) in *G. soli* genome, which may participate in reducing nitric oxide to nitrous oxide, are absent in *G. sulfurreducens*. This foundation indicates that the nitrite metabolism in *G. soli* may be more complex than that in *G. sulfurreducens*.

### Metabolism of electron donors

Glucose cannot be utilized by most members in the genus *Geobacter**.* Although a complete pathway for glycolysis could be reconstructed, *G. sulfurreducens* cannot grow with glucose as an electron donor due to the absence of valid sugar transporter in the genome of *G. sulfurreducens* [27]. To the best of our knowledge, *G. bemidjiensis* was the first *Geobacter* species which can utilize glucose as it possessed a unique glucose/galactose transporter (*gluP* Gbem_3671) belonging to the MFS superfamily [25]. The MFS superfamily is one of the two largest families of membrane transporters, which has a diversity of substrates including simple sugars [28]. In the genome of strain GSS01^T^, besides a complete glycolysis pathway, 8 MFS transporters were found, 7 of which have orthologs in *G. sulfurreducens* and 1 is unique in *G. soli* (SE37_04190, 76 % similarity to that of *Thauera aminoaromatica*). Strain GSS01^T^ was able to grow with glucose as electron donor using Fe(III) citrate as the terminal electron acceptor, and this ability may be attributed to the presence of the unique MFS transporter in *G. soli* genome.

Acetate is expected to be the key electron donor supporting Fe(III) reduction in the *Geobacter* species. Like *G. sulfurreducens*, *G. soli* utilize acetate by two reversible pathways, indicating that acetate may be inefficiently utilized at low concentrations [29]. The first pathway of acetate activation occurs through succinyl-CoA:acetate CoA-transferases (SE37_13685, SE37_00360, and SE37_11235) that convert succinyl-CoA to succinate during oxidation of acetate by the tricarboxylic acid (TCA) cycle [30]. Among the three enzymes, SE37_13685 and SE37_00360 have orthologs in *G. sulfurreducens*, and SE37_11235 is 83 % identical to Gbem_2843 in *G. bemidjiensis*. The second pathway consists of two steps: acetate kinases (SE37_01820 and SE37_14395) convert acetate to acetyl-phosphate, and phosphate acetyltransferase (SE37_01825) converts acetyl-phosphte to acetyl-CoA [30].

Strain GSS01^T^ can grow with pyruvate as an electron donor. The interconvert pyruvate and acetyl-CoA is the central reaction during the pyruvate metabolism. Like other *Geobacteraceae* [25, 30], *G. soli* possesses two sets of genes encoding pyruvate dehydrogenase complexes (SE37_03080, SE37_02045, SE37_02040, and SE37_03100; SE37_03105 and SE37_02035) to irreversibly convert pyruvate to acetyl-CoA. The reverse reaction in *G. soli* from acetyl-CoA is attributed to a homodimeric pyruvate-ferredoxin/flavodoxin oxidoreductase (SE37_14040). In addition to pyruvate, ethanol is another electron donor that strain GSS01^T^ can use but *G. sulfurreducens* cannot. There are two alcohol dehydrogenases (SE37_00690 and SE37_01915) predicted in *G. soli* genome, in which only SE37_00690 has homolog in *G. sulfurreducens* (GSU0573) and SE37_01915 that unique for *G. soli* has 76 % similarity to that in *Vibrio parahaemolyticus*.

Hydrogen is an electron donor utilized by some *Geobacter* such as *G. sulfurreducens**.* In the *G. soli* genome, there are 27 ORFs for hydrogenases, including the orthologs of three large and small subunit [NiFe] hydrogenases (SE37_13920=GSU0122, SE37_13915=GSU0123, SE37_10910=GSU0785, SE37_10925=GSU0782, SE37_03185=GSU2419, and SE37_03190=GSU2418) and two hydrogenase complexes (first complex: SE37_01595 = GSU0739, SE37_01600=GSU0740, SE37_01605=GSU0741, SE37_01610=GSU0742, SE37_01615=GSU0743, SE37_01620 = GSU0745 and possibly SE37_01575=GSU0734; second complex: SE37_01780=GSU2718, SE37_01775=GSU2719, SE37_01770=GSU2720, SE37_01765=GSU2721, SE37_01760=GSU2722) that predicted to participate the hydrogen cycling [31]. In addition, at least two hydrogenases (SE37_02470 and SE37_02475) in *G. soli* genome have no orthologs in *G. sulfurreducens*. This result indicates that *G. soli* may utilize hydrogen as sole electron donor.

Aromatic compounds represent the second most abundant class of natural carbon compounds and many aromatic compounds are major environmental pollutants [32]. Some *Geobacter* species especially *G. metallireducens* have the ability to degrade aromatic compounds [33, 34]. Although there is no complete aromatic compound pathway in the genome of *G. soli*, some genes that may be involved in aromatic compounds degradation are found. For example, 3-hydroxybutyrul-CoA dehydrogenase (SE37_11190, 86 % similarity to Gmet_1717 in *G. metallireducens*), acetyl-CoA acetyltransferase (SE37_11195, 83 % similarity to Gmet_1719 in *G. metallireducens*) , thiolase (SE37_13640), and tautomerase (SE37_07305) are predicted to be involved in the benzoate degradation; one 4Fe-4S ferredoxin (SE37_08830) and six hydrogenase (SE37_10910, SE37_13915, SE37_13920, SE37_10925, SE37_02470 and SE37_02475) are predicted to be involved in nitrotoluene degradation, among which SE37_02470 and SE37_02475 have no ortholog in *G. sulfurreducens*; CoA-transferase (SE37_11150, 85 % similarity to that of *G. metallireducens*) and glutaconate CoA-transferase (SE37_11145, 90 % similarity to Gmet_1708 in *G. metallireducens*) may be involved in styrene degradation, and these enzymes have no orthologs in *G. sulfurreducens*. In addition, other enzymes of acyl-CoA metabolism are predicted from the genome of *G. soli*: acyl-CoA dehydrogenase (SE37_11155, 80 % similarity to Gmet_1710 in *G. metallireducens*; SE37_11180, 86 % similarity to that of *Geoalkalibacter subterraneus*), succinyl-CoA:acetate CoA-transferases (SE37_00360; SE37_11235, 83 % similarity to Gbem_2843 in *G. bemidjiensis*; SE37_13685), acyl-CoA thioesterases (SE37_09325, SE37_09950, SE37_10860, SE37_14445 and SE37_15385), enoyl-CoA hydratases (SE37_15375; SE37_11185, 81 % similarity to Gmet_1716 in *G. metallireducens*), phenylacetate-CoA ligase (SE37_04405, SE37_06045 and SE37_06085) and acyl-CoA synthetase (SE37_06810). The ability to utilize aromatic compounds and other carbon sources may be due to stepwise breakdown of multicarbon organic acids to simpler compounds by these enzymes [29].

## Conclusions

*Geobacter soli* type strain GSS01^T^, isolated from China, can reduce insoluble Fe(III) oxides, such as ferrihydrite, with a variety of electron donors under anaerobic conditions [3]. The insight to the whole genome sequence of strain GSS01^T^ was made based on its ability to reduce electron acceptors with various electron donors. The investigation, especially analysis of the electron transport genes, will be helpful for revealing the mechanism of the extracellular electron transfer of strain GSS01^T^, and further study of the gene-coding sequence may consequently enhance the understanding of the Fe(III) oxides reduction of *Geobacter* genus and even microbial community in anaerobic soils and sediments.

## Additional files

Additional file 1: Table S1. Associated MIGS record. (PDF 191 kb) Additional file 2:
**Amino acid sequence alignment of pilin domain protein SE37_07695 from Geobacter soli GSS01 and those from other bacteria.** Amino acid sequence alignments were generated using the Clustal X (1.8). (PDF 40 kb)
