# Supplementary material for: Genome sequence of a dissimilatory Fe(III)-reducing bacterium Geobacter soli type strain GSS01T
Source: Stand Genomic Sci. 2015 Dec 2;10:118. doi: 10.1186/s40793-015-0117-7 (PMC4667449; doi:10.1186/s40793-015-0117-7)
Supplement: Additional file 1: Table S1. — Associated MIGS record. (PDF 191 kb) [file 40793_2015_117_MOESM1_ESM.pdf]

Table S1. Associated MIGS record

MIGS-ID	Field name	Description
**MIGS-1**	Submit to INSDC/Trace archives	JXBL00000000
**MIGS-2**	MIGS CHECK LIST TYPE	
**MIGS-3**	Project Name	*Geobacter* sp. GSS01 Genome sequencing
**MIGS-4**	Geographic Location	
**4.1**	Latitude	23.37° N
**4.2**	Longitude	112.70° E
**4.3**	Depth	3.5 m beneath the surface
**4.4**	Altitude	11 m
**MIGS-5**	Time of Sample collection	Mar 14, 2013
**MIGS-6**	Habitat (EnvO)	Forest soil
**6.1**	temperature	Mean annual temperature is 21°C
**6.2**	pH	
**6.3**	salinity	
**6.4**	chlorophyll	
**6.5**	conductivity	
**6.6**	light intensity	
**6.7**	dissolved organic carbon (DOC)	
**6.8**	current	
**6.9**	atmospheric data	
**6.10**	density	
**6.11**	alkalinity	
**6.12**	dissolved oxygen	
**6.13**	particulate organic carbon (POC)	
**6.14**	phosphate	
**6.15**	nitrate	
**6.16**	sulfates	
**6.17**	sulfides	
**6.18**	primary production	
**MIGS-7**	Subspecific genetic lineage	Strain GSS01^T
**MIGS-9**	Number of replicons	1
**MIGS-10**	Extrachromosomal elements	0
**MIGS-11**	Estimated Size	3657100 bp
**MIGS-12**	Reference for biomaterial or Genome report	
**MIGS-13**	Source material identifiers	Strain GSS01^T
**MIGS-14**	Known Pathogenicity	Not reported
**MIGS-15**	Biotic Relationship	
**MIGS-16**	Specific Host	
**MIGS-17**	Host specificity or range (taxid)	
**MIGS-18**	Health status of Host	

**MIGS-19**	Trophic Level	Chemeheterotroph
**MIGS-22**	Relationship to Oxygen	Anaerobic
**MIGS-23**	Isolation and Growth conditions	[3]
**MIGS-27**	Nucleic acid preparation	DNA extraction kit (Aidlab)
**MIGS-28**	Library construction	
**28.1**	Library size	Two libraries with insert size 463 bp and 6712 bp
**28.2**	Number of reads	463 bp library: 5118074 reads; 6712 bp library: 2574528 reads
**28.3**	vector	
**MIGS-29**	Sequencing method	Illumina Hiseq 2000
**MIGS-30**	Assembly	
**30.1**	Assembly method	SOAP*denovo* 2.04
**30.2**	estimated error rate	
**30.3**	method of calculation	
**MIGS-31**	Finishing strategy	
**31.1**	Status	High-Quality draft
**31.2**	coverage	165×
**31.3**	contigs	15
**MIGS-32**	Relevant SOPs	
**MIGS-33**	Relevant e-resources	
