# Supplementary material for: Genome sequence of a dissimilatory Fe(III)-reducing bacterium Geobacter soli type strain GSS01T
Source: Stand Genomic Sci. 2015 Dec 2;10:118. doi: 10.1186/s40793-015-0117-7 (PMC4667449; doi:10.1186/s40793-015-0117-7)
Supplement: Additional file 2: — Amino acid sequence alignment of pilin domain protein SE37_07695 from Geobacter soli GSS01 and those from other bacteria. Amino acid sequence alignments were generated using the Clustal X (1.8). (PDF 40 kb) [file 40793_2015_117_MOESM2_ESM.pdf]

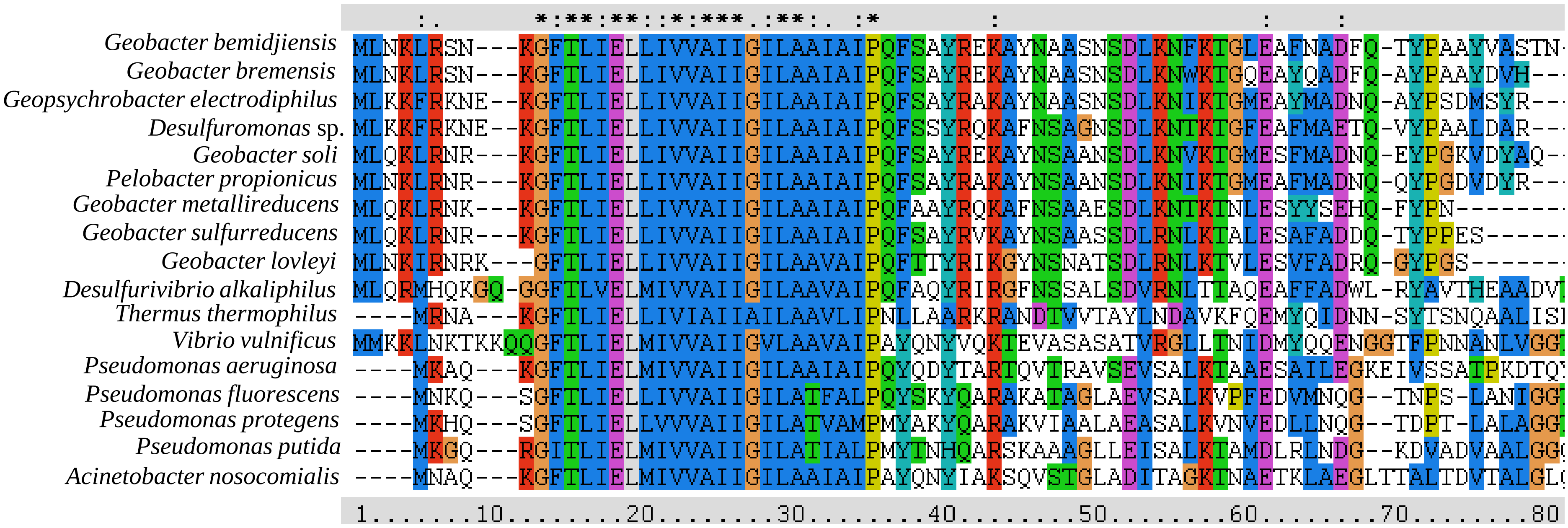

Figure S1 Amino acid sequence alignment of pilin domain protein SE37_07695 from Geobacter soli GSS01 and those from other bacteria. Amino acid sequence alignments were generated using the Clustal X (1.8).

Different amino acid residues were assigned to one of a number of groups (by chemical similarity) and each group was assigned a colour.

The GenBank accession number of the sequences used for the alignment analysis were ACH39598 for *G. bemidji*ensis, WP_026840453 for *G. bremensis*, WP_020676438 for *Geopsychrobacter electrodiphilus*, WP_027714750 for *Desulfuromonas* sp., ABK99270 for *Pelobacter propionicus*, ABB31633 for *G. metallireducens*, WP_049786599 for G. sulfurreducedns, ACD95812 for *G. lovleyi*, ADH86948 for *Desulfurivibrio alkaliphilus*, AAS81202 for *Thermus thermophilus*, ACK37858 for *Vibrio vulnificus*, ABN80239 for *Pseudomonas aeruginosa*, AEV64986 for *Ps. fluorescens*, AAY94501 for *Ps. protegens*, AAN66259 for *Ps. putida*, and CDG73934 for *Acinetobacter nosocomialis.*
